# Synthesis conditions influencing formation of MAPbBr_3_ perovskite nanoparticles prepared by the ligand-assisted precipitation method

**DOI:** 10.1038/s41598-020-72826-6

**Published:** 2020-09-24

**Authors:** Anna Jancik Prochazkova, Markus Clark Scharber, Cigdem Yumusak, Ján Jančík, Jiří Másilko, Oliver Brüggemann, Martin Weiter, Niyazi Serdar Sariciftci, Jozef Krajcovic, Yolanda Salinas, Alexander Kovalenko

**Affiliations:** 1 Linz Institute for Organic Solar Cells (LIOS), Physical Chemistry, Johannes Kepler University Linz, Altenberger Straße 69, 4040 Linz, Austria; 2 Faculty of Chemistry, Materials Research Centre, Brno University of Technology, Purkyňova 118, 61200 Brno, Czech Republic; 3 Institute of Polymer Chemistry, Johannes Kepler University Linz, Altenberger Straße 69, 4040 Linz, Austria

**Keywords:** Materials science, Nanoscience and technology, Optics and photonics

## Abstract

This work reports on an optimized procedure to synthesize methylammonium bromide perovskite nanoparticles. The ligand-assisted precipitation synthetic pathway for preparing nanoparticles is a cost-effective and promising method due to its ease of scalability, affordable equipment requirements and convenient operational temperatures. Nevertheless, there are several parameters that influence the resulting optical properties of the final nanomaterials. Here, the influence of the choice of solvent system, capping agents, temperature during precipitation and ratios of precursor chemicals is described, among other factors. Moreover, the colloidal stability and stability of the precursor solution is studied. All of the above-mentioned parameters were observed to strongly affect the resulting optical properties of the colloidal solutions. Various solvents, dispersion media, and selection of capping agents affected the formation of the perovskite structure, and thus qualitative and quantitative optimization of the synthetic procedure conditions resulted in nanoparticles of different dimensions and optical properties. The emission maxima of the nanoparticles were in the 508–519 nm range due to quantum confinement, as confirmed by transmission electron microscopy. This detailed study allows the selection of the best optimal conditions when using the ligand-assisted precipitation method as a powerful tool to fine-tune nanostructured perovskite features targeted for specific applications.

## Introduction

Nanostructured lead halide perovskite materials have gained significant attention in the last few years mainly because of their potential for application in the field of electronics and photonics. There are several available reports demonstrating their implementation into light emitting diodes (LED)^1–3^, solid-state lasers^4^, solar cells^5^, photodetectors^6^ and optical cooling systems^7^. Especially the photoluminescence quantum yields (PLQY) of up to 100% make these nanostructured perovskite materials very interesting^8^. In addition, their optical properties can be tailored efficiently by tuning the halide composition and stoichiometry^9^ and their size due to quantum confinement effects^10,11^. Depending on the synthetic approach, nanomaterials with defined size and shape can be obtained ranging from nanoplatelets^12^, nanowires^13^, nanorods^14^, nanocubes^15^ to quantum dots^16^. The shape and size can be controlled by changing the reaction conditions and the solvent during hot-injection^17–19^ or ligand-assisted precipitation methods, where also the choice of the capping agents plays a crucial role^20^. Furthermore, perovskite nanoparticles can also be grown within nanopores^21^, or in templates of diblock copolymer micelles^22^.

Most synthetic approaches for MAPbBr_3_ perovskite nanoparticles are based on the room-temperature ligand-assisted precipitation method where the capping agents are used for the formation of nanostructures^23^. This method is convenient and cost-effective due to its mild equipment requirements and moderate operating temperatures. Another benefit of the ligand-assisted precipitation method is that the product of the reaction is a colloidal dispersion that can be easily processed by spin coating^24^, centrifugal casting^8^ or ink-jet printing^25^. However, there are several important parameters which need to be controlled during this ligand-assisted precipitation technique. Hence, the resulting final properties of the nanomaterials depend on the precipitation temperature^26^, the choice of solvent system^27^, the choice of capping agents^8,27^ and their concentration^28^ and on the water concentration in the precursor solutions^11^. However, a detailed understanding of the interplay of all these parameters is currently missing.

Various types of capping agents have been applied recently to stabilize perovskite nanomaterials. Among them, ligands containing primary amine groups along with carboxylic acids are used for stopping the crystal growth, providing higher stability in the colloidal solutions^18^. Commonly used amines are long-chain primary amines^18,23^ and zwitterion-based capping agents^29^. In fact, our group previously demonstrated that the use of bio-inspired capping agents, such as amino acids^27^ and cyclopeptides^30^ can also control the nanoparticle growth. Besides, promising stabilizing properties were also found for adamantane-based capping agents^8,31^, where colloidal solutions exhibit a PLQY up to 100%.

Herein, this manuscript provides a comprehensive study of the parameters that influence nanoparticle formation when applying the ligand-assisted precipitation method. We optimized the properties of adamantane-1-amine (AdNH_2_) stabilized MAPbBr_3_ perovskite nanoparticles (PNP) by varying temperature, concentration and solvents. We report the optical and physical properties along with the temporal stability of precursor solutions and colloids.

## Results and discussion

**Figure 1:**
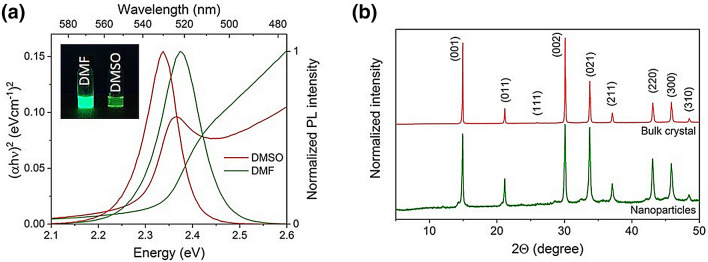
(**a**) Comparison of colloidal solutions in toluene prepared from two different precursor solutions (in DMF and DMSO)—the inset shows photos of colloidal solutions under UV irradiation (excitation wavelength 366 nm); (**b**) Powder XRD spectra of PNP prepared from DMF precursor solution compared to the spectra of bulk MAPbBr_3_ single crystal.

In general, perovskite precursors are soluble in highly polar aprotic solvents such as *N,N*-dimethylformamide (DMF), dimethylsulfoxide (DMSO) and γ-butyrolactone (GBL)^32^. Encouraged by previous experiments, we tested DMSO^30^ and DMF^27^ as solvents for the preparation of precursor solutions. The precursors were soluble in both DMSO and DMF. For both solvents, a transparent clear precursor solution was obtained, see Figure S1. The colloidal solutions prepared from precursors in the DMF and DMSO exhibited green emission under a UV-lamp (Fig. 1). Figure 1a shows the comparison between the absorption and emission spectra of the dispersions prepared in toluene from the DMF and DMSO precursor solutions. The emission maximum of the colloidal solutions prepared from DMF and DMSO precursor solutions was of 522 and 530 nm and the photoluminescence quantum yield (PLQY) was of 43% and 2%, respectively (see Table 1). The red-shifted emission maximum and the decrease of PLQY of the sample from precursor solution prepared in DMSO can be related to the retarded crystallization process during the precipitation. Due to its strong binding to the lead precursor^33^, the capping agents cannot be involved in the crystal growth control and in the PNP stabilization. The better optical properties of the colloidal solution prepared from the DMF precursor solution led to using DMF as a precursor solvent for the further studies. As a first step, the robustness of the PNP synthesis was tested by preparing DMF-based precursor solutions followed by the precipitation in toluene as described above. Colloidal solutions were obtained with an emission maximum of 521 ± 2 nm, a PLQY of 48 ± 7% and a band gap in the range 2.32–2.34 eV suggesting a good reproducibility of the PNP synthesis procedure.

**Table 1:** Comparison of optical properties of colloidal solutions prepared from precursor solutions in DMF or in DMSO.

Coordinating solvent	Emission maximum (nm)	FWHM (nm)	PLQY (%)	Band gap (eV)
DMF	521 ± 2	22 ± 1	48 ± 7	2.33 ± 1
DMSO	530 ± 2	18 ± 1	3 ± 2	2.30 ± 1

The prepared PNP were collected by centrifugation of the colloidal solutions. The resulting material was dried at ambient conditions overnight to ensure the complete evaporation of the solvent traces. Using X-ray powder diffraction (XRD) the perovskite crystalline structure was confirmed by comparing PNP diffractogram with bulk MAPbBr_3_ diffraction patterns^33^ (Fig. 1b). Peaks positioned at 14.90°, 21.14°, 26.02°, 30.07°, 33.72°, 37.08°, 43.05°, 45.83° and 48.42° 2θ correspond to the reflections from the planes (001), (011), (111), (002), (021), (211), (220), (300), and (310), respectively^34^. The broadening of the diffraction peaks observed in the PNP spectrum results from the small crystal volume of the individual nanoparticles^27^.

Next, the influence of the precursor solution concentration in the resulting colloidal solutions on the PNP formation was studied. For that, samples were prepared by precipitating precursor solution in defined ratios in toluene at 3 °C and their optical properties were determined (see data collected in Table 2). The concentration of PbBr_2_ was 0.027, 0.054, 0.081, 0.14 and 0.27 mmol L^−1^ in the resulting toluene dispersion and the concentrations of MABr, AdNH_2_ and HeA were adjusted accordingly to keep their ratio of 1.1, 0.8 and 9.5 with respect to PbBr_2_. As a result, the obtained colloidal solutions showed very similar optical properties (Table 2). Therefore, it can be assumed that the ratio between the precursor solution and the precipitation medium has no significant effect on the formation of PNP. Nevertheless, with higher concentration of nanoparticles formed in the precipitation medium, the optical density of the resulting dispersion was observed to increase (Figure S2a). Following, detected optical densities were of 0.15, 0.24, 0.35, 0.56 and 0.52 at the excitation wavelengths of 405 nm in the integrating sphere for colloidal solutions prepared with the concentration of 0.027, 0.054, 0.081, 0.14 and 0.27 mmol L^−1^ of PbBr_2_ in the resulting toluene dispersion, respectively. It must be emphasized that at high concentrations, the recorded PLQY values may be inaccurate due to strong self-absorption of the colloidal dispersion^35^. Consequently, the concentration of 0.054 mmol L^−1^ of PbBr_2_ in the precipitation medium was used for further experiments because of the accurate optical density of 0.24 at the excitation wavelength of 405 nm.

**Table 2:** Comparison of optical properties of colloidal solutions prepared with different ratios of precursor solution; the concentration of PbBr_2_ was recalculated for the whole volume of the colloidal solution.

Concentration of PbBr_2_ (mmol/L)	Emission maximum (nm)	FWHM (nm)	PLQY (%)	Band gap (eV)
0.027	521	22	41	2.33
0.054	520	22	43	2.34
0.081	520	21	41	2.34
0.14	520	22	45	2.33
0.27	521	21	62	2.33

As reported previously^26^, there is a significant effect of the precipitation temperature on the resulting properties of the colloidal solutions. Therefore, in order to evaluate the influence of the temperature during the precipitation on the resulting optical properties of colloidal solutions, temperatures from − 5 to 80 °C were varied by using an ice or an oil bath. In all cases, a colour change was detected during the precipitation and the colloidal solutions were formed, as seen in Figure S3. The temperature changes from − 5 up to 40 °C brought a negligible shift in emission spectra, while the PLQY was affected significantly. The highest PLQY of ca. 48% with the emission maximum of 521 nm was obtained for samples prepared at 3 °C. Interestingly, when the precipitation temperature was 60 and 80 °C, a red-shift of the emission maxima and a strong decrease of PLQY was clearly noticed (Table 3). Along with the emission maximum at ca. 528 nm, the optical properties of the colloidal solution were similar to the optical properties of a polycrystalline MAPbBr_3_ film where the emission maximum is 530 nm and a line width of 23 nm (FWHM) was reported^36^. Hence, it could be assumed that increasing the precipitation temperature enhances the tendency to form the perovskite lattice, and in this case, the crystallization could have occurred too promptly to be controlled by the capping agents.

**Table 3:** Comparison of optical properties of colloidal solutions prepared at different temperatures.

Temperature (°C)	Emission maximum (nm)	FWHM (nm)	PLQY (%)	Band gap (eV)
− 5	521 ± 2	22 ± 1	36 ± 6	2.32 ± 1
3	521 ± 2	22 ± 1	48 ± 7	2.33 ± 1
13	520 ± 2	22 ± 1	34 ± 6	2.35 ± 1
25	523 ± 2	23 ± 1	20 ± 5	2.34 ± 1
40	523 ± 2	22 ± 1	20 ± 5	2.33 ± 1
60	528 ± 2	19 ± 1	7 ± 2	2.30 ± 1
80	528 ± 2	21 ± 1	3 ± 2	2.29 ± 1

**Figure 2:**
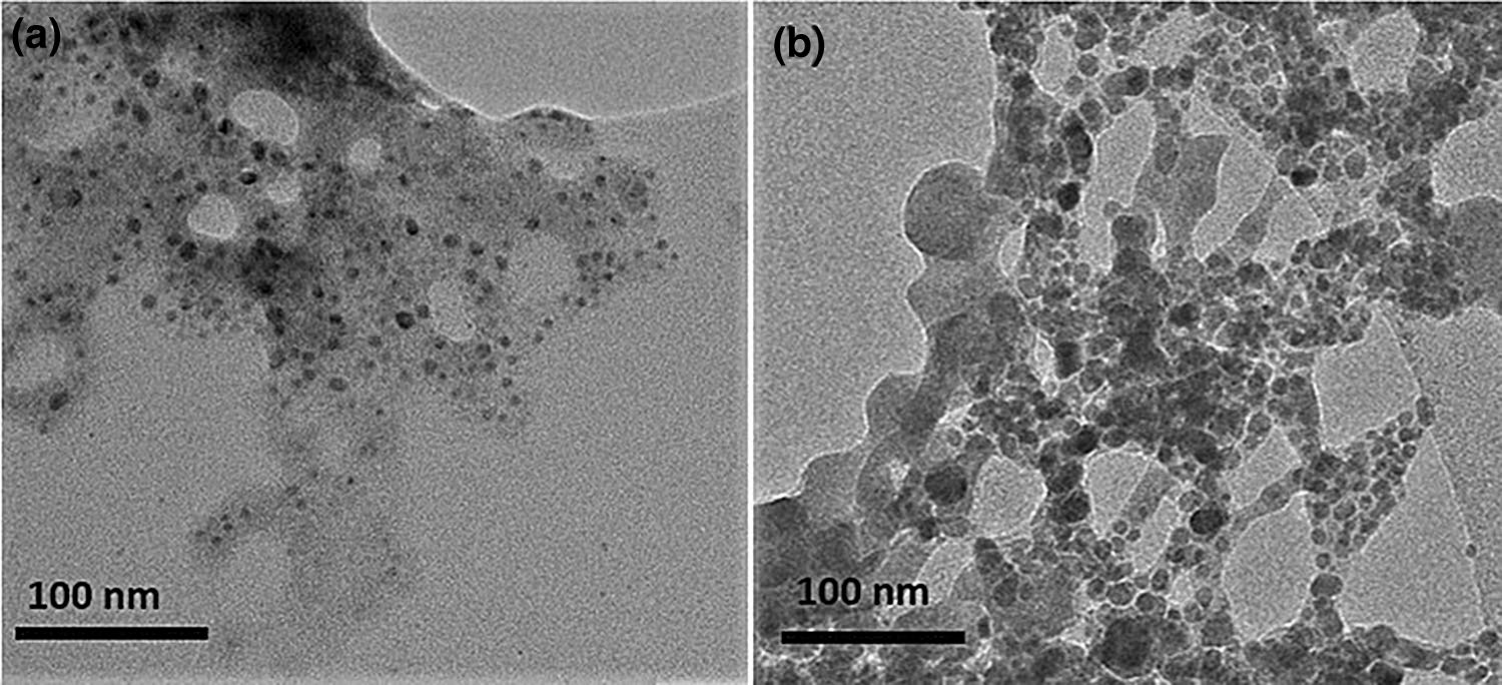
TEM images of PNP. The samples were prepared from the crude colloidal solution (**a**) and from the thrice-washed colloidal solution (**b**).

Furthermore, the influence of washing PNP with toluene on the optical properties of colloidal solutions was investigated. A colloidal solution was prepared by precipitating precursor solution in toluene in a volume ratio of 0.002 under cold conditions (3–4 °C) in order to keep the optimal ratio of precursor and antisolvent for the optical characterization. The sample was washed up to three times, thus four samples were obtained from one batch in total—crude colloidal solution and then colloidal solutions once, twice and three times washed. The volume of added toluene for the redispersion was always set to keep the original concentration of PNP. Remarkably, no significant changes in emission spectra were detected when washing the colloidal solutions. However, a strong decrease in PLQY was detected after the purification procedure, the PLQY typically dropped to 40–60% of the original value. A typical example is shown in Table 4, where a decrease in PLQY was observed, from 54 to 30%, after the colloidal solution was washed three times. Hence, the influence of the washing procedure on the resulting nanoparticles’ size, shape and possible formation of aggregates was analysed by transmission electron microscopy (TEM). TEM images were taken of crude and thrice-washed samples dropped on a TEM grid for comparison. No significant change was observed between both samples. In fact, in both cases, the nanoparticles formed aggregates, while more individual nanoparticles were visualized from the sample prepared with crude colloidal solution, see Fig. 2. In TEM images it can be seen that individual nanoparticles are covered with an amorphous shell. The shell is most likely formed from the surface ligands that appeared during the nanoparticle formation. However, due to the Van der Waals intermolecular interaction the shell is in equilibrium at a nanoparticle surface, therefore it is not expected to be removed during the washing steps. Apparently, the washing steps did not have any significant effects on the morphology of individual nanoparticles and the spherical shape and highly crystalline structure were not affected, as demonstrated in Figure S3, concluding that repeated washing does not affect the morphology of the PNPs. Even so, unwashed particles showed a typical diameter of 8.5 ± 1.8 nm, whereas a diameter of 9.3 ± 2.0 nm was observed after 3 times of washing with toluene. This could be attributed to the possible removal of the capping agents from the PNP surface^37^ which could reduce the final colloidal stability of the PNP. Moreover, the thinner organic shell formed around the nanoparticles could increase the probability of non-radiative transitions between them, thus decreasing the PLQY values.

**Table 4:** Optical properties of crude and washed colloidal solutions.

Washing step	Emission maximum (nm)	FWHM (nm)	PLQY (%)
Crude	522	23	54
Washed 1x	522	23	43
Washed 2x	523	23	35
Washed 3x	523	23	30

From an application point of view, it is important to test solvent systems suitable for PNP processing, since the possibility of preparing colloidal solutions in different precipitation media may increase the applicability of these materials^38^. Therefore, different precipitation media were tested during the preparation of the precursor solutions and chlorobenzene, n-hexane, cyclohexane, 1-octadecene, acetonitrile, tetrahydrofuran, diethylether, chloroform, xylene, acetone and toluene were selected. Remarkably, only chlorobenzene, diethylether, chloroform, xylene and toluene supported the formation of a colloidal solution exhibiting green light emission under UV irradiation (Figure S4). These colloidal solutions were further characterized by optical spectroscopy techniques to evaluate the changes in optical properties. In particular, the use of chloroform as an antisolvent resulted in the formation of colloidal solutions with the most blue-shifted emission spectrum with a maximum of 509 nm, in agreement with our previously reported work^27^. Besides, a strong photoluminescence quenching effect^39^ was observed in the case of the sample prepared in chloroform where the PLQY was found <1%. On the other hand, the use of toluene resulted in the formation of colloidal solution with the emission maximum of 521 nm with a PLQY of ca. 48%. All recorded optical properties of the samples are summarized in Table 5.

**Table 5:** Optical properties of colloidal solutions prepared in different solvents.

Solvent	Emission maximum (nm)	FWHM (nm)	PLQY (%)	Band gap (eV)
Chloroform	509 ± 2	23 ± 1	> 1	2.37 ± 1
Diethylether	518 ± 3	23 ± 1	33 ± 6	2.37 ± 1
Chlorobenzene	519 ± 2	20 ± 1	34 ± 5	2.36 ± 1
Xylene	520 ± 2	20 ± 1	34 ± 5	2.33 ± 1
Toluene	521 ± 2	22 ± 1	48 ± 7	2.33 ± 1

**Figure 3:**
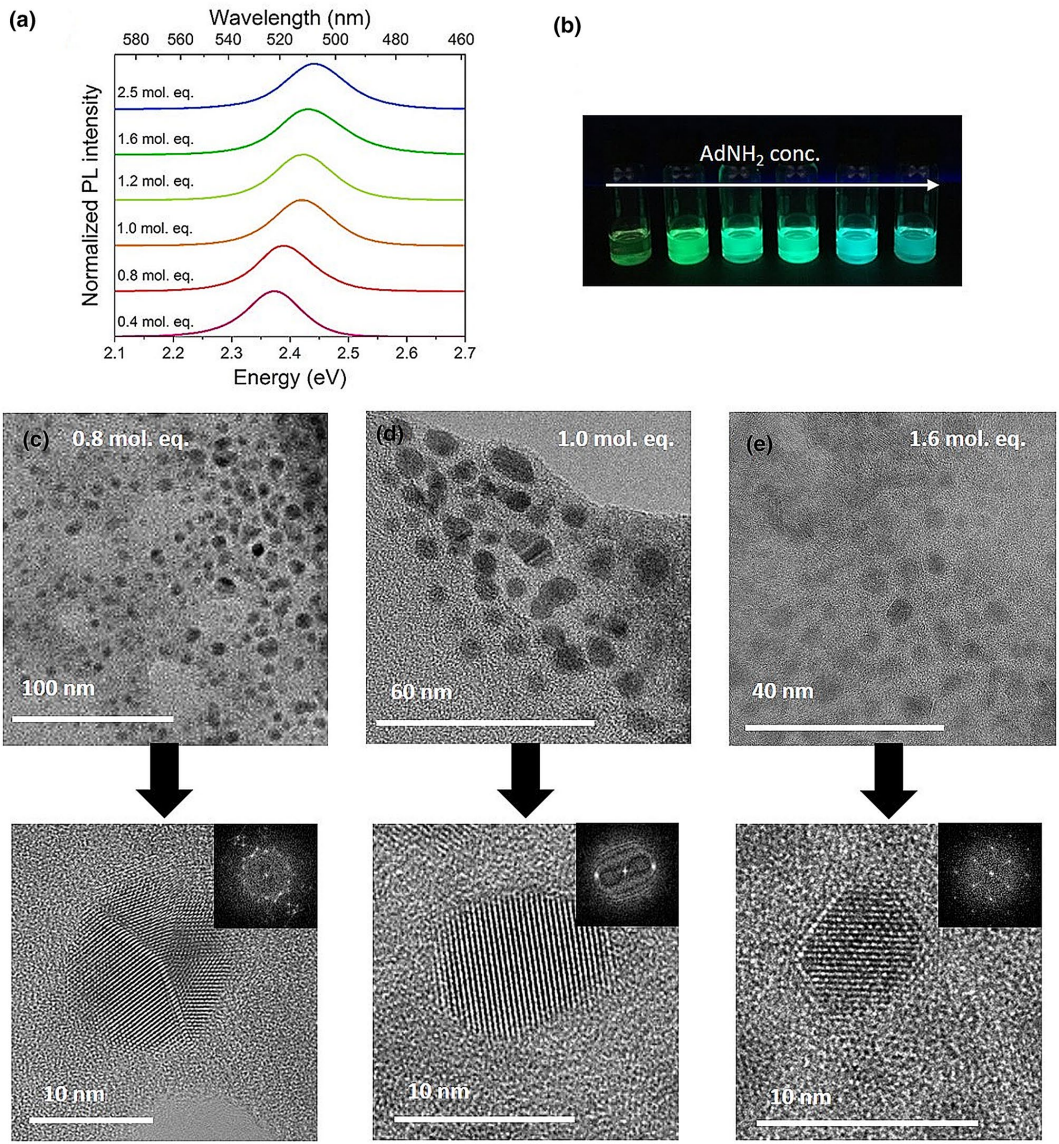
Varying concentrations of AdNH_2_ in precursor solution and the influence on optical properties and the size of nanoparticles: (**a**) Optical characterization of the colloidal solutions; (**b**) photo of the colloidal solutions under UV irradiation (excitation wavelength 366 nm); (**c**–**e**) TEM images of PNP prepared from precursors with 0.8, 1.0 and 1.6 molar equivalents of AdNH_2_ with respect to PbBr_2_, respectively, and zoom to individual nanoparticles with FFT images in the insets.

The ratio of the precursor chemicals was also supposed to influence the resulting optical properties^28^. For that reason, different precursor solutions with varying concentration of MABr, AdNH_2_ and HeA were prepared while the concentration of PbBr_2_ was kept constant. The emission maxima shifted to higher wavelengths with increasing MABr concentration in the precursor solution (see Table 6 and Figure S7). As expected, PLQY was affected by the MABr concentration changes and the highest PLQY of 40% was observed for a PbBr_2_:MABr ratio of 1:1.1. A decrease in PLQY detected with increasing MABr concentration was attributed to replacing AdNH_2_ by methylammonium ions on the nanoparticles’ surface. Here, the probability of non-radiative transitions between the particles could increase because of thinning the organic shell. Alternatively, increasing the concentration of AdNH_2_ in the precursor solutions led to blue-shifted emission and to an increase in the PLQY. Also, by keeping the molar ratio of AdNH_2_ at 0.4 and 2.5 with respect to PbBr_2_, emission maxima of ca. 523 and 509 nm were detected, respectively, and simultaneously, broadening of the emission spectra was observed (Fig. 3a–b). Here, as expected, the amount of the amino-capping agent had a significant effect on PLQY, which increased with the concentration of AdNH_2_. The maximum detected PLQY was 70% when 1.6 molar equivalents of AdNH_2_ were used with respect to PbBr_2_. A further increase in the concentration of the amino-capping agent to a 2.5 molar ratio with respect to PbBr_2_ did not lead to a further blue-shift of the photoluminescence, but in a decreased PLQY to about 55%. Regarding to the results, the ratios between precursor chemicals have a crucial effect on the resulting PLQY. Hence, fine tuning of the ratios leads to a significant increase in PLQY. From this point of view, quite big deviations can be explained by this observation—even negligible changes in the precursor chemicals concentration can cause a significant change in PLQY.

**Table 6:** Optical properties of colloidal solutions where the PNP were prepared with different precursors ratios.

	Emission maximum (nm)	FWHM (nm)	PLQY (%)	Band gap (eV)
**MABr ratio**				
1.9	528 ± 2	20 ± 1	18 ± 6	2.27 ± 1
1.1*	521 ± 2	22 ± 1	48 ± 7	2.33 ± 1
0.9	519 ± 2	23 ± 1	36 ± 6	2.33 ± 1
**AdNH**_**2**_** ratio**				
2.5	509 ± 2	31 ± 2	55 ± 6	2.46 ± 1
1.6	508 ± 2	27 ± 2	68 ± 5	2.45 ± 1
1.2	511 ± 2	27 ± 1	67 ± 7	2.44 ± 1
1.0	513 ± 2	26 ± 1	61 ± 6	2.44 ± 1
0.8*	521 ± 2	22 ± 1	48 ± 7	2.33 ± 1
0.4	523 ± 2	23 ± 1	8 ± 4	2.32 ± 1
**HeA ratio**				
19	521 ± 2	22 ± 1	53 ± 6	2.35 ± 1
9.5*	521 ± 2	22 ± 1	48 ± 6	2.33 ± 1
4.8	522 ± 2	21 ± 1	29 ± 5	2.33 ± 1
2.4	523 ± 2	21 ± 1	36 ± 6	2.34 ± 1

*Standard ratios for the PNP preparations which are described in the experimental section.

The blue-shift in emission spectra connected to the increasing the AdNH_2_ concentration in the precursor solution can be attributed to the formation of smaller nanoparticles. To confirm this assumption, precursor solutions containing 0.8, 1.0 and 1.6 molar equivalents of AdNH_2_ with respect to PbBr_2_ were selected to prepare PNPs for the TEM characterization (Fig. 3c–e). In all samples, spherical and highly crystalline nanoparticles were observed, with average sizes of 8.5 ± 1.8 nm, 6.2 ± 0.4 nm and 4.5 ± 0.6 nm, respective to the concentration. For comparison, the Bohr radius calculated for the MAPbBr_3_ perovskite materials was 4.7 nm^40^. Interestingly, the concentration of HeA in the precursor solutions did not show any significant influence on the emission maxima, but it had a strong effect on PLQY (Figure S7). Changing the molar ratio of HeA with respect to PbBr_2_ from 2.4 to 19 resulted in the formation of colloidal solutions with emission maxima between ca. 523 and 521 nm, and PLQYs of ca. 36% and 53%, (see Table 6). This effect may be explained by the possible formation of a stronger organic shell surrounding the individual nanoparticles and a reduction of surface defects leading to a more radiative recombination.

**Figure 4:**
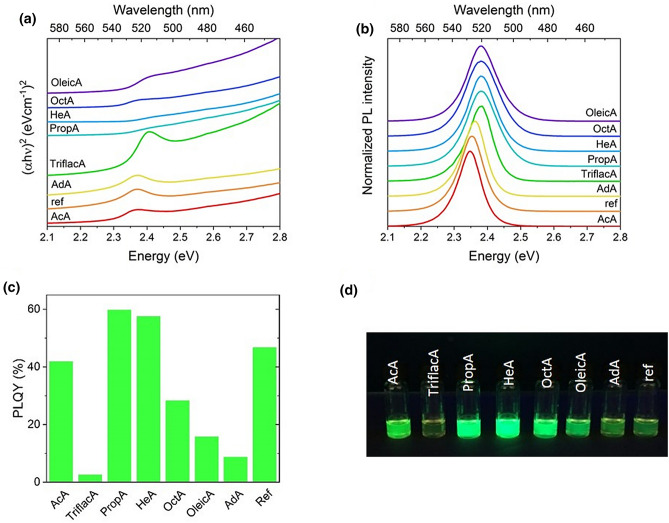
Optical properties of colloidal solutions of PNP with different carboxylic acids as capping agents: (**a**) UV–Vis spectra, (**b**) PL spectra, (**c**) PLQY values and (**d**) photos of the colloidal solutions under UV irradiation (excitation wavelength 366 nm).

With respect to a previously reported study^8^, different carboxylic acids were selected to stabilize the PNP and to evaluate whether the optical properties can be further improved. For that, a fixed ratio of precursors was set to 1:1.1:0.9:9.5 for PbBr_2_:MABr:AdNH_2_:carboxylic acid. Here, the selected carboxylic acids were acetic acid (AcA), trifluoroacetic acid (TriflacA), propanoic acid (PropA), hexanoic acid (HeA), n-octanoic acid (OctA), oleic acid (OleicA) and adamantanecarboxylic acid (AdA). The optical properties of the corresponding colloidal solutions are summarized in Fig. 4 and Table 7. For comparison, a colloidal solution without addition of carboxylic acid was prepared (called reference sample, ref).

**Table 7:** Comparison of optical properties of PNP colloidal solutions with different capping agents.

Carboxylic acid	Emission maximum (nm)	FWHM (nm)	PLQY (%)	Band gap (eV)
AcA	528 ± 2	19 ± 1	42 ± 6	2.30 ± 1
TriflacA	520 ± 2	20 ± 1	3 ± 2	2.33 ± 1
PropA	521 ± 2	25 ± 1	58 ± 7	2.32 ± 1
HeA	521 ± 2	22 ± 1	51 ± 7	2.32 ± 1
OctA	523 ± 2	26 ± 1	35 ± 5	2.31 ± 1
OleicA	521 ± 2	24 ± 1	16 ± 5	2.33 ± 1
AdA	524 ± 2	19 ± 1	7 ± 4	2.27 ± 1
Reference	528 ± 2	20 ± 1	45 ± 6	2.26 ± 1

**Figure 5:**
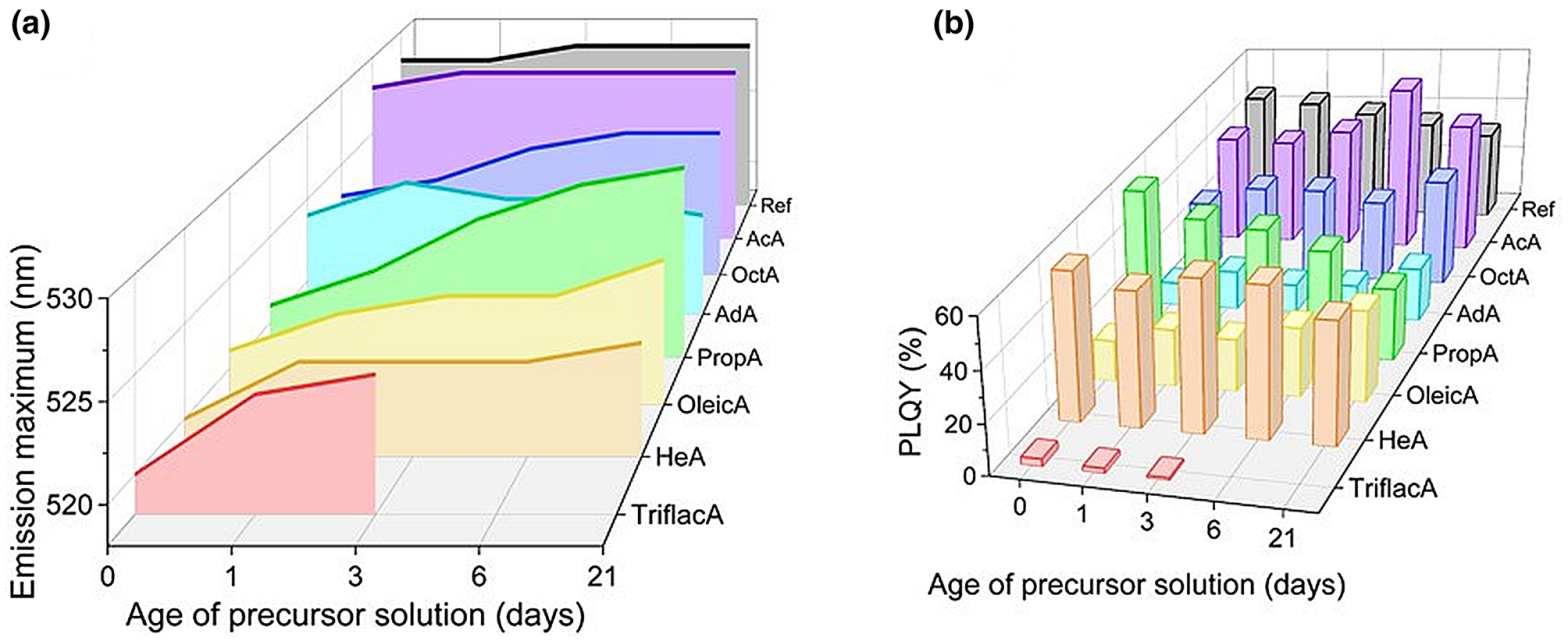
Colloidal stability of PNP prepared with different carboxylic acids as surfactants: (**a**) emission maxima and (**b**) PLQY monitoring over a period of 21 days.

The stability of colloidal solution prepared for precursor solutions with different carboxylic acids was also further tested. Colloidal solutions were prepared after stirring the precursor solutions for one day and the optical properties of the resulting colloidal solutions were studied over a period of 21 days. It was observed that all the samples tended to precipitate within that time, but nevertheless the solid material was easy to redisperse under a sonication bath. As shown in Fig. 5a, a red-shift was observed over time in all samples except for the sample where PNPs were stabilized by AdNH_2_ and AdA. It must be noted that the precursor solution containing AdA as a carboxylic acid containing a capping agent formed a cloudy solution, while all the other precursor solutions were clear. This behaviour could be related to the low solubility of the precursors in the solution. For the TriflacA-based solution, the photoluminescence was no longer detectable after three days.

The selection of the surfactant was also further investigated, it was observed that if an appropriate surfactant is chosen, the emission maximum is blue-shifted and PLQY can be increased. For example, the choice of PropA leads to the formation of colloidal solution with an emission maximum of 521 nm with a PLQY of ca. 58%, and a band gap of 2.32 eV. Furthermore, the use of the carboxylic acid improved the colloidal stability and the PLQY of the PNP stabilized by HeA was lowered from initial 58% to 49% over the period of 21 days. The use of any carboxylic acid for the stabilization was noticed to decrease the PLQY from 47 to 33% in the same period. As comparison, a colloidal solution with no carboxylic acid as surfactant was prepared and apparently, it was possible to prepare PNP colloidal solutions without any further stabilization by surfactants. The colloidal solution showed an emission maximum at 528 nm, a PLQY of 45% and a band gap of 2.28 eV.

In order to fully characterize the prepared PNP, X-ray photoelectron spectroscopy (XPS) and Fourier transform infrared (FT-IR) spectroscopy were employed to study the ligands (AdNH_2_ and HeA) binding to the PNP surface and the complex formation in precursor solutions. XPS revealed the binding energies of the core electrons providing an information about the chemical environment of the individual atoms comprising the nanoparticles^41^. Two symmetric peaks at 138.3 eV and 143.2 eV were detected in the Pb 4f. high resolution XPS spectra pointing out to Pb 4f7/2 and Pb 4f5/2 signals, respectively (see Figure S6) with a characteristic spin–orbit splitting of 4.9 eV^42,43^. Simultaneously, the presence of Br 3d5/2 and Br 3d3/2 was proved by detecting peaks at 68.2 eV and 69.2 eV in the Br 3d spectrum^44^. Both Pb and Br XPS spectra confirm the presence of the perovskite-based material in the sample. On the other hand, the N 1s XPS spectrum showed one peak with the centre at 401.8 eV pointing out to the presence of the methylammonium salt. Nevertheless, it can be simultaneously associated with the ammonium salt of AdNH_2_ proving the charged character of the primary ammonium group in AdNH_2_ molecules stabilizing the PNP^43^. Three peaks were deconvoluted in the C 1s XPS spectrum. Peaks with the centres at 285.0, 286.2 and 289.2 eV can be attributed to C–C/C–H bonds of adamantyl-moiety and the HeA side chain, then to the C–N bond of the amino groups^43^ and finally to the C–O bond of the carboxylic acid group of HeA, respectively^45^. The latter could support an assumption about the deprotonated nature of the carboxylic group because of exhibiting one signal due to the delocalized character of the negative charge^46^. This can be also supported by recording only one peak with the maximum at 533.0 eV in the O 1s XPS spectrum. The quantification of the perovskite components showed ratios of 2.7 and 1.2 for Br/Pb and N/Pb, respectively, which is in accordance with previously reported results^8^.

**Figure 6:**
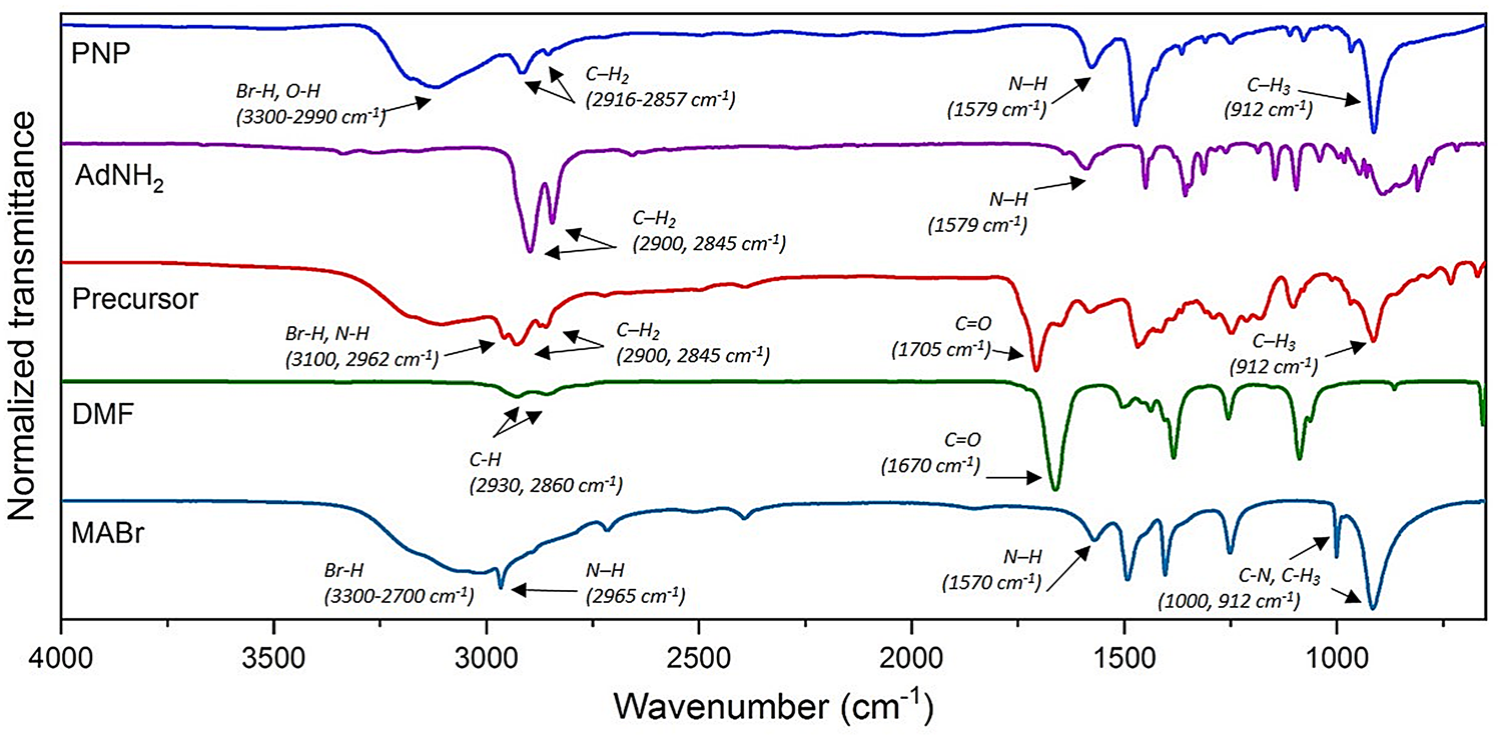
FT-IR spectra of PNP stabilized with 0.8 mol equation of AdNH_2_ within the precursor containing PbBr_2_, MABr and HeA in solvent DMF.

Additionally, FT-IR spectroscopy was used to detect the presence of the capping ligands AdNH_2_ and HeA decorating the surface of the PNP precipitated from the precursor solution prepared in DMF (see Fig. 3). The three main characteristic peaks of MABr were easily detected at 3300–2700 cm^−1^ (broad signal assigned to the Br–H), at 1000 and 912 cm^−1^ (narrow and medium signals) assigned to C–H rocking bonds ^47,48^. In the bare solvent, the C = O detected at 1670 cm^−1^ was shifted in the precursor to a lower wavenumber (1650 cm^−1^) due to the interaction between PbBr_2_ and MABr^49^. Signals at 3300–2990 and 912 cm^−1^ detected in PNP spectrum could correspond to the vibrations of the methylammonium ions in the surrounding of PbBr_3_^−^. Bands at 2900 and 2845 cm^−1^ related to –CH_2_ groups from AdNH_2_ appeared at a higher wavenumber in PNP (2916 and 2857 cm^−1^, respectively), which was an indication of nanoparticles’ surface passivation. In the precursor, the signals at 2930 and 2855 cm^−1^ were attributed to the alkyl groups and the signal detected at 1705 cm^−1^ was assigned to the C = O stretching band from the carboxylic acid group presented in the ligand hexanoic acid, introduced here as a stabilizer. Furthermore, the signal at 1595 cm^−1^ from the amine of the capping agent AdNH_2_ was shifted to a lower wavenumber 1579 cm^−1^ attributed to the possible coordination with the PbBr_3_^−^ from the amino groups^27^. The aforesaid is in agreement with preferential stabilization of nanoparticles with the selected ligands (Fig. 6).

## Conclusion

In the present work, a complex comparative study of non-template PNP preparation via the ligand-assisted precipitation technique was carried out. Various parameters were controlled during the preparation of PNP stabilized by AdNH_2_ and their influence on the resulting optical properties was evaluated. It was found that the temperature during the precipitation has a crucial effect on the optical properties, a red-shift in emission spectra and a decrease in PLQY occurs with increasing temperature, which was the result of larger nanoparticles. We assume that at temperatures higher than 40 °C the perovskite lattice forms too fast to be efficiently stabilized by the capping agents. At the same time, the choice of the precipitation medium influences the resulting optical properties. As suitable precipitation media, nonpolar solvents such as chlorobenzene, chloroform, toluene and xylene were evaluated. Also, diethylether as a polar aprotic solvent which is known as an antisolvent for perovskite materials^50^ was proven to be suitable for PNP preparation. Strong nonpolar aliphatic solvents, such as hexane, cyclohexane and 1-octadecene, and strong polar solvents such as acetonitrile, acetone and tetrahydrofurane were not able to induce perovskite lattice formation. The best precipitation medium was found to be toluene by means of higher PLQY in colloidal solution, but the use of different solvent systems could broaden the applicability of functional devices. In addition, the ratio of precursor chemicals also significantly influenced the optical properties of the nanoparticles due to quantum confinement effects. Especially, increasing the concentration of AdNH_2_ leads to more efficient control of the crystal growth during the preparation, thus, smaller PNP may be obtained can take place. Moreover, different carboxylic acids were tested as surfactants to evaluate the influence of the length and the bulkiness of their side chain on the resulting optical properties, colloidal stability and on the stability of precursor solution. In agreement with previous reports^8^, too bulky and long-chain carboxylic acids such as AdA and OleicA were not suitable for surface passivation, probably due to the sterical hindrances. However, high PLQY were detected when short chain carboxylic acids such as PropA and HeA were used as a capping agent together with AdNH_2_. We strongly believe that this work will help to better understand the preparation of PNP colloidal solutions and thus to effectively tailor not only the optical properties but also the composition (i.e. capping agents, used precipitation medium) for the desired application.

## Materials and methods

### Chemicals

Lead (II) bromide (PbBr_2_, 99.999%) and methylammonium bromide (MABr, 98%) were purchased from Sigma Aldrich. Adamantyl-1-amine (AdNH_2_) and adamantanecarboxylic acid (AdA, 99.6%) were obtained from Provisco CS. Hexanoic acid (HeA, 98%) and n-Octanoic (OctA, > 98%) acid were purchased from TCI. Oleic acid (OleicA, min. 70%) was purchased from Lachner. Glacial acetic acid (AcA, 99–100%) was purchased from J. T. Baker. Trifluoroacetic acid (TriflacA, 99.5 + %) was purchased from Alfa Aesar. Propionic acid (PropA, > 99.5%) and 1-Octadecene (90%) were provided by Sigma Aldrich. *N,N*-dimethylformamide (DMF), xylene (a mixture of isomers), acetone, diethylether, cyclohexane, chlorobenzene, chloroform and toluene were of reagent grade, n-hexane and acetonitrile (ACN) were of HPLC grade, all provided from VWR. All chemicals were used as received.

### General methods

#### Nanoparticle synthesis

Following the previous report by S. González-Carrero *et al*^8^*.* adamantane-1-amine (AdNH_2_) was used for the stabilization of perovskite nanoparticles (PNP). The synthetic procedure was modified in order to optimize the PNP preparation. Initially, the ratio of precursor chemicals was 1:1.1:0.8:9.5 for PbBr_2_:MABr:AdNH_2_:HeA and as a coordinating solvent *N,N*-dimethylformamide (DMF) was selected. The resulting concentration of precursors was then 0.027, 0.030, 0.022 and 0.26 mol L^−1^ for PbBr_2_, MABr, AdNH_2_, and HeA, respectively. All the precursor solutions were prepared 24 h in advance, in order to favour the complex formation while being stirred. The precipitation of 20 μl of the precursor solution was performed in 10 mL of toluene (precursor solution and toluene were in a volume ratio of 0.002) which acted as a non-coordinating non-polar solvent under cold conditions (ice bath at 3–4 °C), while being stirred. Washing steps were performed by centrifuging the crude colloidal solutions (at 5000 rpm during 10 min) and the resulting solid material was redispersed by sonication in toluene.

#### General methods

Ultraviolet–visible (UV–Vis) and fluorescence spectroscopy were used to determine the optical properties of the prepared PNP. UV–Vis spectroscopy was carried out with a Lambda 1050 UV/Vis/NIR spectrometer (PerkinElmer). Photoluminescence (PL) spectra were measured on a QuantaMaster 40 from Photon Technology International. The PLQY of colloidal PNP suspensions were measured using a Hamamatsu C9920-03 absolute quantum yield spectrometer. For all PLQY measurements, an excitation wavelength of 405 nm was chosen. Fourier-transform infrared (FTIR) spectra of precursor solutions were recorded on a Perkin-Elmer Spectrum 100. The precursor solutions were measured by using an ATR technique at ambient conditions. Transmission electron microscopy (TEM) images were obtained with a Jeol JEM-2200 microscope using Holey Carbon film 300 Mesh copper grids. All sample grids were previously treated for 5 min in a Jeol EC-52000IC Ion cleaner before the TEM measurement. Powder X-ray diffraction (XRD) measurements were performed on an Empyrean (PANalytical) diffractometer using Cu Kα (1.540598 Å) radiation. The measurement parameters were as follows: tube current 30 mA and voltage 40 kV; scan axis gonio; step size 0.013°2θ; time per step 96 s. Solid samples for powder XRD measurements were prepared by precipitating the precursor solution in toluene in a volume ratio of 0.01 at 3–4 °C. Solid material was collected after the centrifugation (5000 rpm for 10 min) followed by careful washing with toluene (approx. 3 mL). PNP were dried at room temperature for 12 h. X-ray photoelectron spectroscopy (XPS) was employed in order to investigate the binding energy of a core-level electron of an atom in the nanoparticles. The XPS sputter depth profiles were performed using a Thetaprobe XPS system (Thermo Scientific, UK), which was controlled and operated by the Avantage software package from the system manufacturer. The device was equipped with a monochromated Al K_α_ X-Ray source (hν = 1486.6 eV) and a dual flood gun for neutralizing the surface charges. The X-ray spot on the sample surface exhibited a diameter of 400 µm. The sputtering was performed by using an Ar^+^ ion gun. The survey spectra were recorded with 200 eV of pass energy and with a binding energy step of 1 eV, while for high-resolution spectra a pass energy of 20 eV and step of 0.05 eV was used. The sample for X-ray photoelectron spectroscopy (XPS) was prepared by centrifugal casting, as reported^8^. The colloidal solution was prepared by precipitating precursor solution in toluene in a volume ratio of 0.002 at 3–4 °C in a centrifugation tube. A glass substrate (1 × 1 cm) was placed on the bottom of the tube and the sample was centrifuged (at 5000 rpm for 10 min). The supernatant was carefully discharged, and the film formed on the substrate was carefully washed with toluene (approx. 3 mL). The prepared film was dried and stored in a glove box before the measurement.

## Supplementary information

Supplementary information
