# Supplementary material for: Synthesis conditions influencing formation of MAPbBr3 perovskite nanoparticles prepared by the ligand-assisted precipitation method
Source: Sci Rep. 2020 Sep 24;10:15720. doi: 10.1038/s41598-020-72826-6 (PMC7518261; doi:10.1038/s41598-020-72826-6)
Supplement: Supplementary file 1 — Supplementary information [file 41598_2020_72826_MOESM1_ESM.docx]

**Supporting information**

**Synthesis conditions influencing formation of MAPbBr_3_ perovskite nanoparticles prepared by the ligand-assisted precipitation method**

Anna Jancik Prochazkova,^1,2*^ Markus Clark Scharber,^1^ Cigdem Yumusak,^1^ Ján Jančík,^2^ Jiří Másilko,^2^ Oliver Brüggemann,^3^ Martin Weiter,^2^ Niyazi Serdar Sariciftci,^1^ Jozef Krajcovic,^2^ Yolanda Salinas^3^ and Alexander Kovalenko^1,2^

^1^Linz Institute for Organic Solar Cells (LIOS), Physical Chemistry, Johannes Kepler University Linz, Altenberger Straße 69, 4040 Linz, Austria

^2^Faculty of Chemistry, Materials Research Centre, Brno University of Technology, Purkyňova 118, 61200 Brno, Czech Republic

^3^Institute of Polymer Chemistry, Johannes Kepler University Linz, Altenberger Straße 69, 4040 Linz, Austria

*Corresponding author

Email: anna@roproch.cz


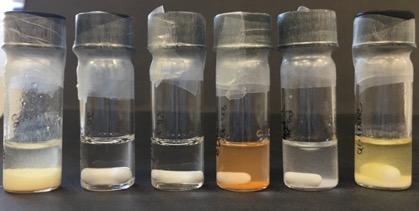


Figure S1: Precursor solutions prepared in DMSO and DMF from left to right in ambient light.


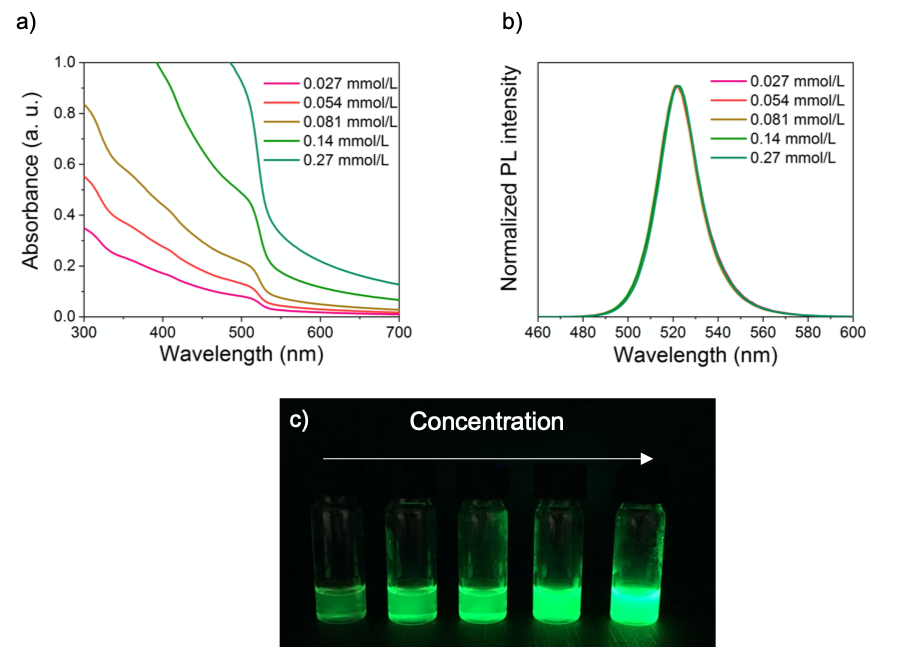


Figure S2: Study of samples prepared with different concentration of PbBr_2_ in precipitation medium (toluene), a) UV-Vis spectra, b) normalized PL spectra, c) photo of colloidal solutions with corresponding concentration of PbBr_2_ under UV irradiation (excitation wavelength 366 nm)


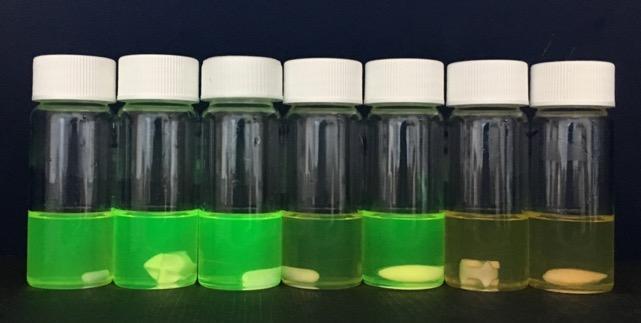


Figure S3: Colloidal solutions prepared by precipitating a precursor solution in toluene at different temperatures under UV irradiation. From left to right the temperatures applied were -5, 2, 13, 25, 40, 60 and 80 °C.


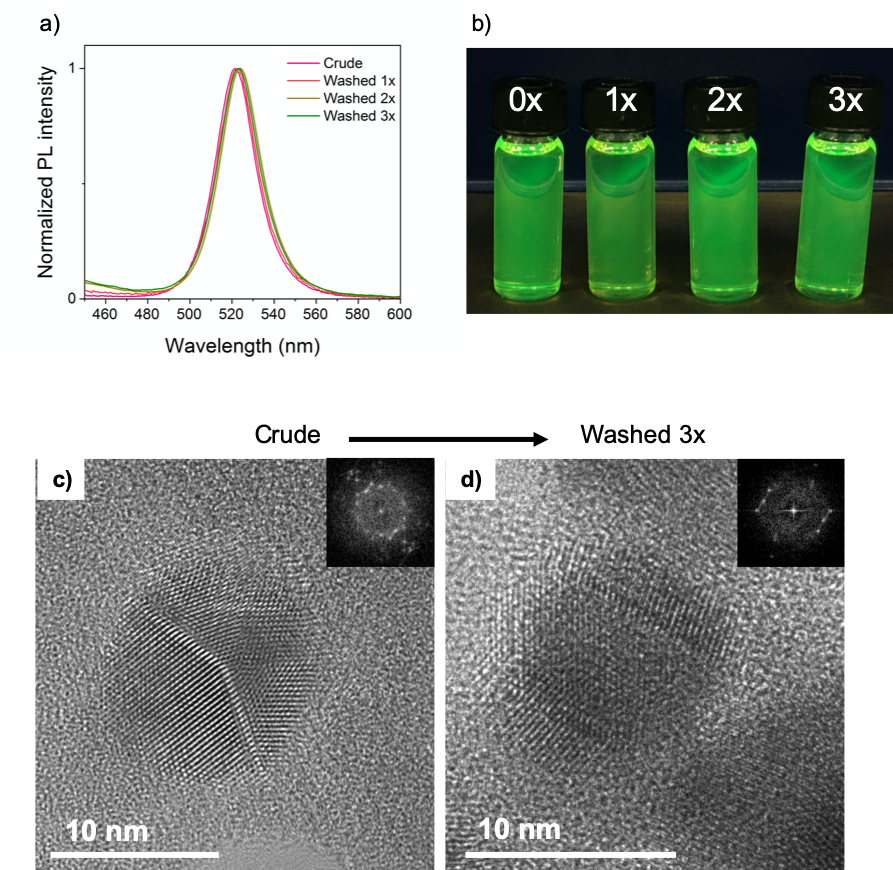


Figure S4: Influence of washing colloidal solutions on optical properties, a) emission spectra of crude, one time, two times and three times washed colloidal solution, b) photos of the corresponding colloidal solutions, c) d) TEM images of crude PNP and of PNP after 3 washing steps, respectively, FFT images included in the insets.


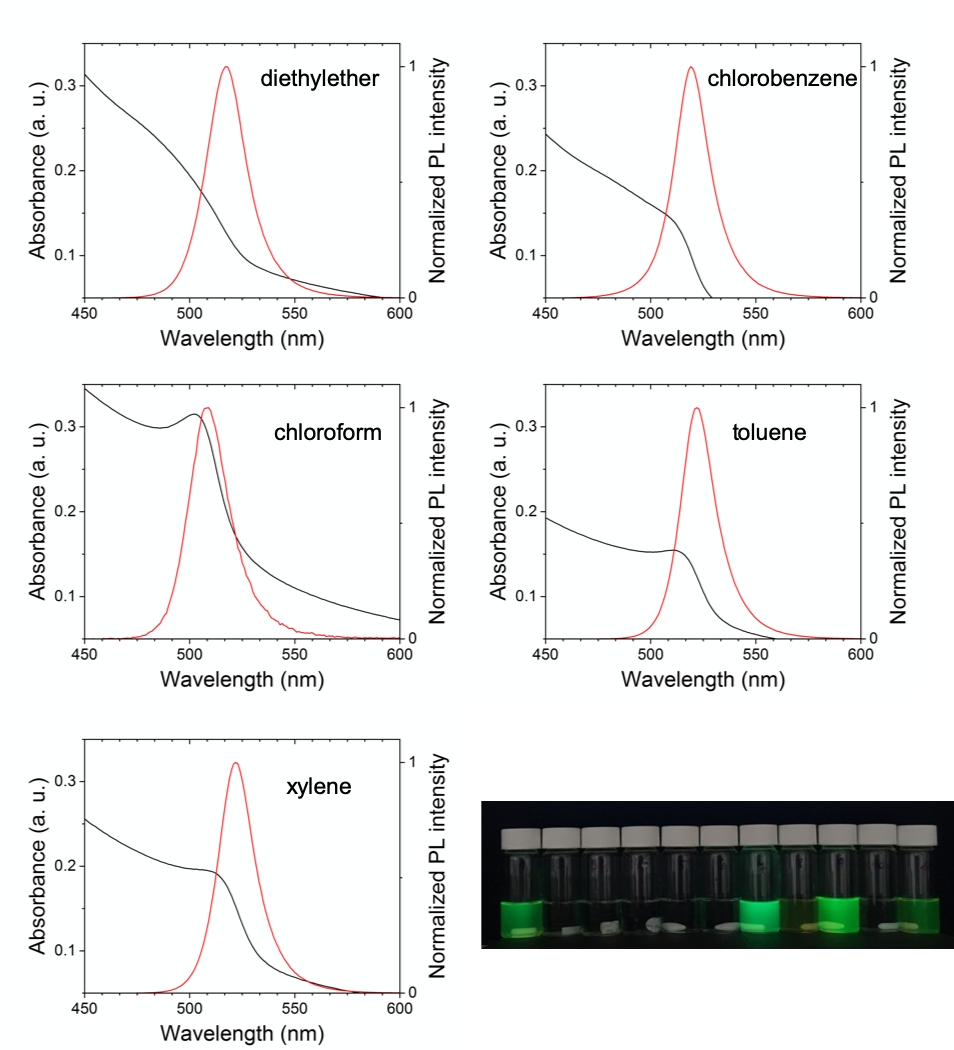


Figure S5: Optical characterization of PNPs colloidal solutions prepared in different precipitation media, chlorobenzene, n-hexane, cyclohexane, 1-octadecene, acetonitrile, tetrahydrofuran, diethylether, chloroform, xylene, acetone and toluene and the solutions’ image in the vials taken under UV irradiation from left to right, respectively.


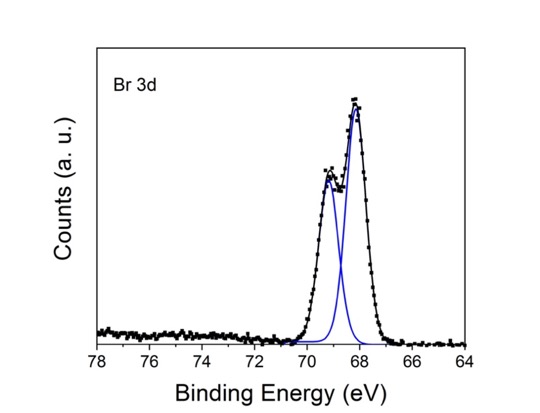

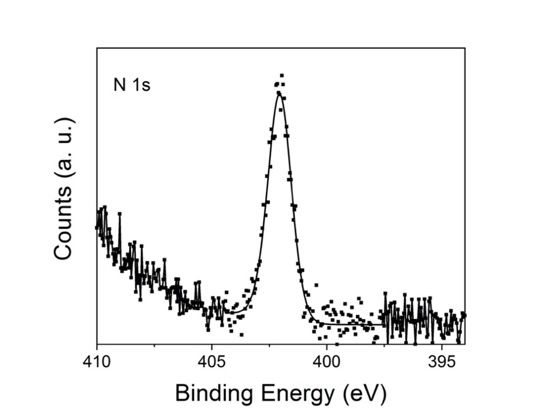

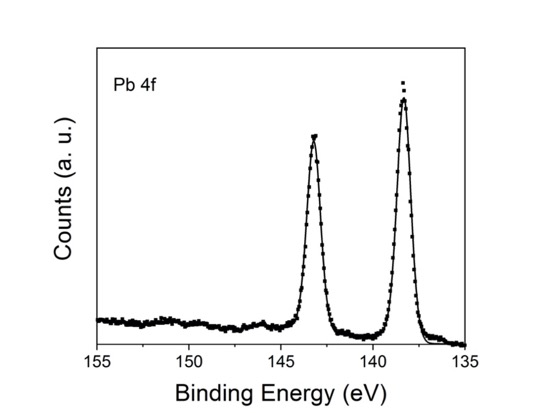

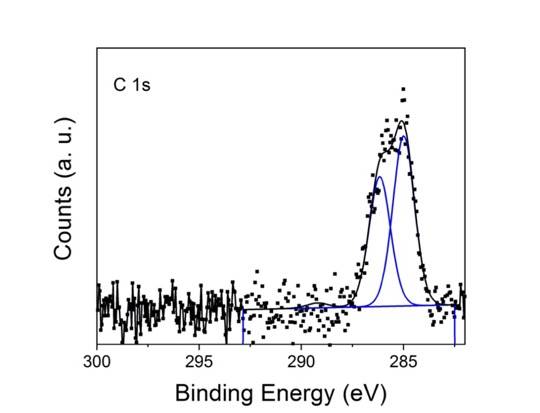

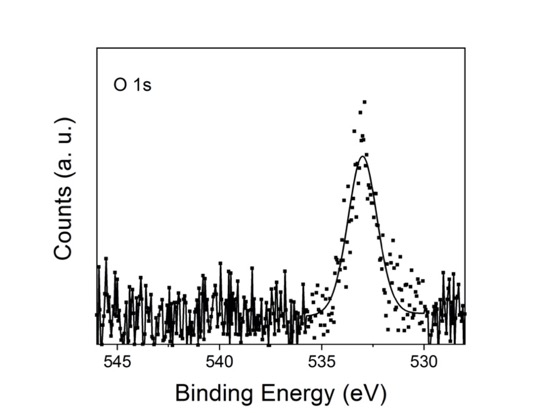

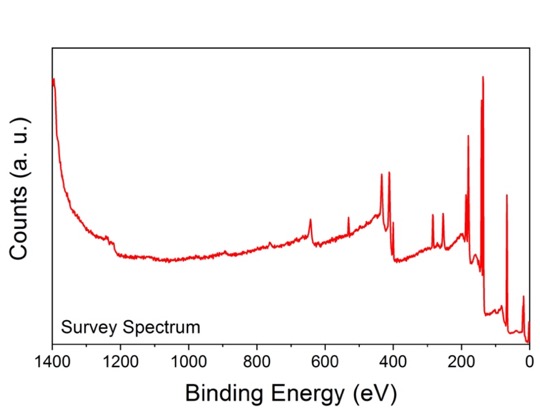


Pb 5d

Br 3d

Pb 4f

Br 3p

Br 3s

C 1s

N 1s

Pb 4d5

Pb 4d3

O 1s

Pb 4p3

Pb 4p1

Pb 4s

Figure S6: XPS analysis of PNP.


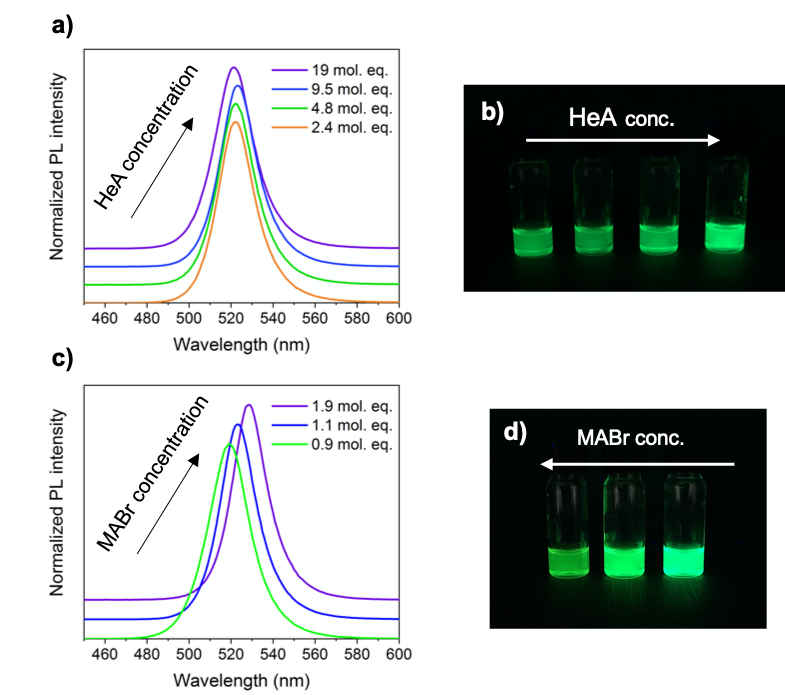


Figure S7: Colloidal solutions with different precursors ratio, a) HeA concentration modifications and resulting PL spectra, b) photos of the vials containing the corresponding colloidal solutions prepared from precursor solutions with different HeA concentration under UV irradiation, c) MABr concentration modifications and resulting PL spectra, d) photo of the vials containing the corresponding colloidal solutions prepared from precursor solutions with different MABr concentration.

**Study of the stability of the precursor solutions**

In order to better understand the stability precursor solutions, different colloidal solutions were prepared from the same precursor solutions after 1, 2, 4, 7 and 22 days stirring at room temperature under ambient conditions. The vials containing the precursor solutions were sealed with parafilm to avoid contamination. Figure S8 shows the changes in emission maxima and in PLQY values upon the precursor solutions storage. The emission maxima slightly shift to higher energies upon longer storage. PNP stabilized by AdNH_2_ and PropA exhibited emission maximum 521 nm when precipitated from 1 day old precursor solution, whereas, when 22 days old precursor solution was used for the colloidal solution preparation, the emission maximum was of 499 nm. The band gaps were increasing correspondingly, for the already mentioned sample shifted from 2.31 to 2.44 eV. Regarding to PLQY values, the PLQY decreased with increasing age of the precursor solution as expected, varying from 60 to 27 %.


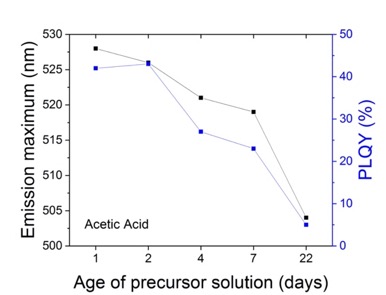

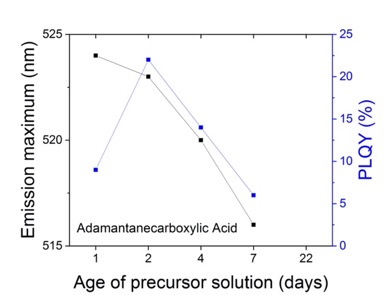

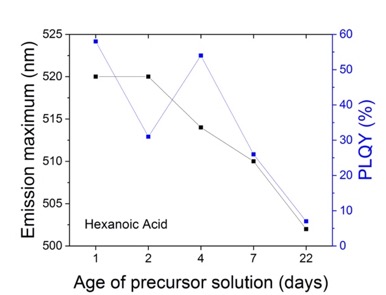

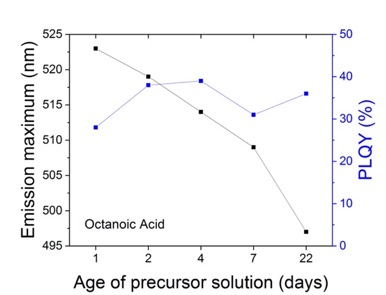

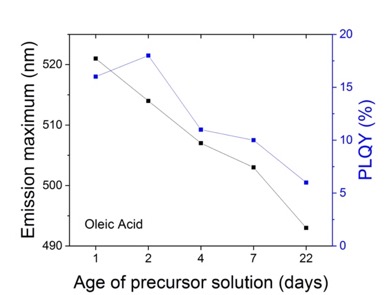

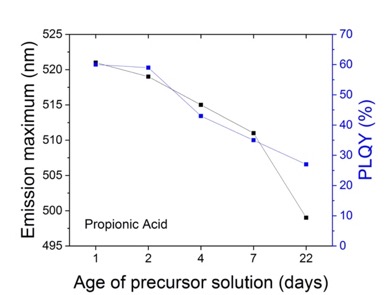

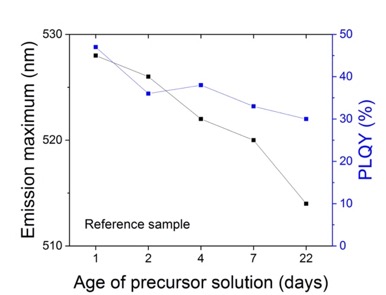

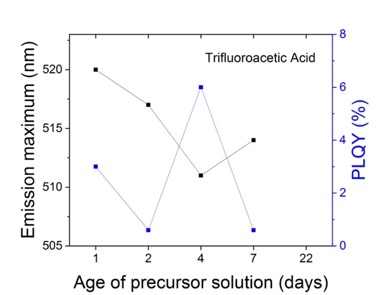


Figure S8: Influence of precursor solution aging time on the final optical properties of colloidal solutions prepared with different carboxylic acids as surfactants.
